# The Interleukin-20 Cytokines in Intestinal Diseases

**DOI:** 10.3389/fimmu.2018.01373

**Published:** 2018-06-18

**Authors:** Jan Hendrik Niess, Petr Hruz, Tanay Kaymak

**Affiliations:** ^1^Department of Biomedicine, University of Basel, Basel, Switzerland; ^2^Department of Gastroenterology and Hepatology, University Hospital of Basel, Basel, Switzerland

**Keywords:** Interleukin-20 family, macrophages, eosinophilic esophagitis, Crohn’s disease, ulcerative colitis, colorectal cancer

## Abstract

Autoimmune/inflammatory intestinal diseases, such as Crohn’s disease and ulcerative colitis, infectious gastrointestinal diseases, and gastrointestinal cancers, such as colorectal cancer, are worldwide a significant health problem. Intercellular communication and direct contact with the environment as the microbiota colonizes the gastrointestinal surface facilitates these diseases. Cytokines mediate the intercellular communication to maintain the equilibrium between host and environment and to regulate immune responses. One cytokine family that exchange information between immune cells and epithelial cells is the IL-20 cytokine family which includes the cytokines IL-19, IL-20, IL-22, IL-24, and IL-26. These cytokines share common receptor subunits and signaling pathways. IL-22 is the most intensively studied cytokine within this family in contexts of gastrointestinal disease, but the importance of other family members is more and more appreciated. In this review, the potential function of IL-20 cytokines concerning gastrointestinal conditions is discussed.

## The IL-20 Cytokine Subfamily within the IL-10 Cytokine Family

**Figure 1:**
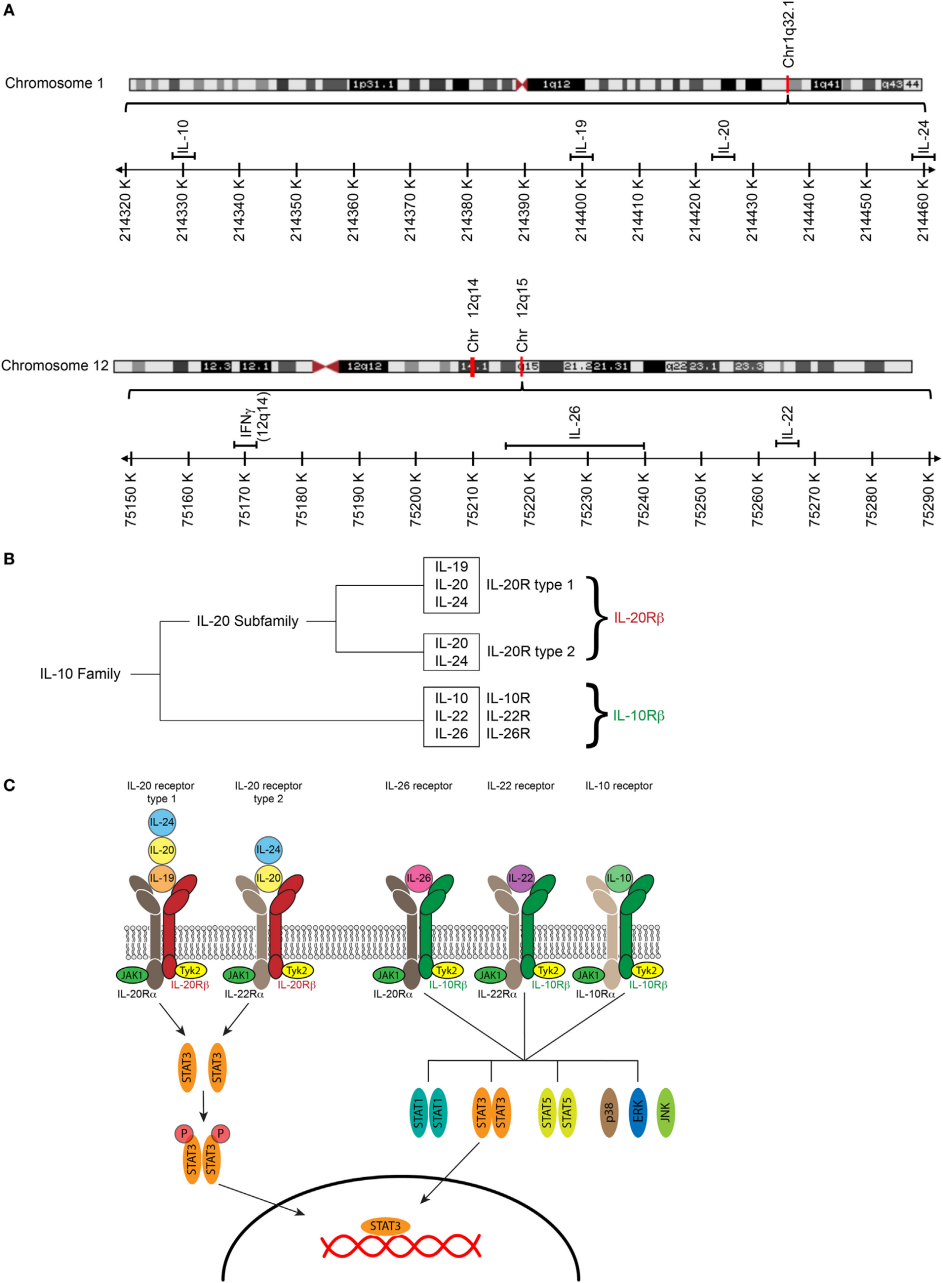
The IL-20 cytokines and their receptors. **(A)** Maps of human chromosome 1 and human chromosome 12 showing close proximity of *IL10, IL19, Il20*, and *IL24* on chromosome 1 and *IL22, IL26*, and *IFNG* on chromosome 12. Structures of respective chromosomes were retrieved from the NCBI genome viewer. **(B)** The IL-20 cytokines IL-19, IL-20, and IL-24 cluster in a distinct evolutionary branch within the IL-10. **(C)** IL-10 receptors are heterodimers consisting of an α and a β -chain. The IL-20 cytokines IL-19, IL-20, and IL-24 signal through the type I IL-20 receptor (IL-20R); IL-20 and IL-24 but not IL-19 can also signal though the type II IL-20R. The IL-26 receptor, the IL-22 receptor, and the IL-10 receptor have the IL-10Rβ-chain in common. IL-10Rβ pairs with IL-20Rα to form the IL-26 receptor, with IL-22Rα to form the IL-22 receptor, and with IL-10Rα to form the IL-10 receptor. Cytokine binding to their receptors leads to an activation of the JAK/STAT pathway [adapted Ref. (3, 4)].

The IL-20 subfamily that includes the cytokines IL-19, IL-20, IL-22, IL-24, and IL-26, is part of the IL-10 cytokine family, which further includes the cytokines IL-10, IL-28, and IL-29. All members of the IL-10 cytokine family signal through heterodimeric receptors composed of an α-chain and β-chain (Figure 1). Members of the IL-20 cytokine subfamily signal through a receptor that consists of at least one IL-20 receptor (IL-20R) chain. IL-20Rβ can either pair with IL-20Rα forming the type I IL-20R, which acts as a receptor for IL-19, IL-20, and IL-24. Furthermore, IL-20Rβ forms with IL-22Rα the type II IL-20R through which IL-20 and IL-24 can signal (1). IL-26, which is only present in humans and not in mice, signals through the IL-26 receptor that consists of IL-20Rα and IL-10Rβ (2). Moreover, IL-22Rα and IL-10Rβ form the IL-22 receptor for IL-22 (1).

The best-characterized cytokine of the IL-20 cytokine family is IL-22 (3, 4). IL-22 is produced by T cells and innate lymphoid cells and induces the production of antimicrobial peptides and mucus by epithelial cells. The other members of the IL-20 subfamily are less characterized concerning their importance in mucosal immunity. Because the expression pattern of IL-20R chains differs between tissues, IL-20 cytokine subfamily members may have different functions in different tissues.

In this review, we will summarize the expression pattern of IL-20Rs in different gastrointestinal tissues and discuss their potential function for intestinal diseases.

## Expression of IL-20R Along the Gastrointestinal Tract

### IL-22 Receptor (IL-22Rα + IL-10Rβ)

IL-22 binds to the IL-22 receptor expressed by intestinal epithelial cells to protect the intestine from damage and to support regeneration by inducing the expression of chemokines involved in cellular mobility, facilitating the expression of the antibacterial lectins RegIIIβ and RegIIIγ and inducing the expression of mucins after STAT3 activation (5). Therefore, the IL-22 receptor is highly expressed in the entire gastrointestinal tract (6, 7) (Table 1).

**Table 1:** Expression of IL-20Rβ, IL-20Rα, and IL-22Rα in intestinal tissues, liver, epidermis, and mesenteric lymph nodes, and cellular source of IL-20 family cytokines.

	Receptor subunits				Cytokines			
Tissue	IL-20Rβ	IL-20Rα	IL-22Rα	Cellular source	IL-19	IL-20	IL-24	IL-22
Distal colon	++	+	+++	Monocytes	+++	+++	+++	−
Proximal colon	++	+	+++	Macrophages	+++	+++	+++	−
Ileum	++	−	+++	Keratinocytes	++	++	++	−
Jejunum	++	−	+++	Epithelial cells	++	++	++	−
Duodenum	++	−	+++	Fibroblasts	+	+	+	+
Glandular stomach	++	−	+++	T cells	−	−	+	+++
Forestomach/esophagus	+++	++	+++	B cells	+	−	−	−
Liver	++	−	++	NK cells	−	−	−	++
Mesenteric lymph nodes	++	−	+	Innate lymphoid cells type 3	−	−	−	++
Epidermis	+++	+++	+++	Dendritic cells	−	+	−	−

*+++, very frequent; ++, frequent; +, present; −, not present*.

### IL-22 Binding Protein (IL-22bp Also Known as IL-22Rα2)

Interleukin-22 binding protein is a secreted soluble, monomeric protein that binds IL-22 and competes with IL-22R for IL-22. IL-22bp can hence inhibit the activity of IL-22 (8).

### Type I IL-20R (IL-20Rα + IL-20Rβ)

In contrast to the IL-22 receptor, the expression of the type I IL-20R is much lower in the distal and proximal colon of mice (6, 7, 9). Increased expression of IL-20Rα, which also signals through STAT3 (10), in colitis models can be explained by infiltration of the inflamed colonic lamina propria with monocyte-derived macrophages that express IL-20Rα (7). IL-20Rα is not expressed in the in the small intestine and the glandular stomach (7) but IL-20Rα is expressed in the forestomach/esophagus of mice with squamous epithelium and the epidermis (7). Thus, the type I IL-20R is mainly expressed at mucosal surfaced with squamous epithelium.

### Type II IL-20R (IL-20Rβ + IL-22Rα)

The type II IL-20R, which signals through STAT3 (10), is expressed in the colon and the small intestine at higher levels than the type I IL-20R but at lower levels than the IL-22 receptor (7). The type I IL-20R serves as a receptor for IL-19, IL-20, and IL-24, while the type II IL-20R is only a receptor for IL-20 and IL-24, but not for IL-19 (3). Signaling through the type I and type II IL-20R may have redundant actions, because different ligands bind to different receptors with different expression patterns of the individual receptor chains. Studies with genetically manipulated mice lacking every single cytokine and each receptor chain are required to delineate this system further.

### IL-26 Receptor (IL-20Rα + IL-10Rβ)

The IL-26 receptor, which signals through STAT1 and STAT3, is not expressed in the small intestine and glandular stomach but is present in the esophagus and also in the colon at low levels, but its contribution to diseases of the esophagus and the colon is unknown.

## Cellular Sources of IL-20 Cytokine Family Members in the Gastrointestinal Tract

### IL-22

Initially, CD11c + mononuclear phagocytes have been identified as the cellular source of IL-22 (6). Now, type 3 innate lymphoid cells (ILC3) (11), CD4 T cells (12), γδ T cells (13), and CD1d-restricted T cells, such as NKT cells (14) are significant sources of IL-22 in the gastrointestinal tract, where IL-22 contributes to regeneration and host defense.

### IL-19

Monocytes (15, 16), macrophages (17), keratinocytes (18), synovial fibroblasts (19), B cells (20), and airway epithelial cells (21) produce IL-19. *In situ* hybridization of mouse colonic tissues demonstrated that F4/80 + macrophages and some intestinal epithelial cells produce IL-19 (7). Analysis of an Il19-tdTomato reporter mouse line confirmed that intestinal macrophages produce IL-19 after stimulation with LPS (7), whereas B cells did not produce IL-19 in un-manipulated Il19-tdTomato reporter mice. Because fate-mapping is not possible in this reporter mouse line, the analysis of Il19-tdTomato reporter mice cannot exclude the possibility that a given cell once expressed IL-19 in its history.

### IL-20

Monocytes (16), macrophages (22), keratinocytes (23), and dendritic cells secretes IL-20. Furthermore, IL-1, IL-8, IL-17, and TNFα induce IL-20 expression in keratinocytes (24). In the colon, the expression of IL-20 is low in un-manipulated mice and also during acute dextran Sodium Sulfate (DSS) colitis. Thus, the cellular source of IL-20 in the gut is not well defined.

### IL-24

Monocytes (16), macrophages (25), endothelial cells (16), keratinocytes (23), melanocytes (26), and subepithelial myofibroblasts (27) produce IL-24. In Th2 cells, the transcription factors STAT6 and GATA3 regulate the expression of IL-24 (28). Furthermore, optimal production of IL-24 by macrophages requires type I IFN signaling and co-stimulation through the IL-4/STAT6 pathway, whereas in NK cells optimal IL-24 production requires type I IFNs and STAT4 (29). In IBD patients, colonic subepithelial myofibroblasts produce IL-24 in response to IL-1β, IL-17, and LPS (27), but the importance of IL-24 for the development of IBD needs to be further studied.

### IL-26

Th17 cells (30), NK cells (27), fibroblasts (27), and macrophages (27) secrete IL-26. In T cells, IL-1β in combination with IL-23 and presence of low concentration of TGFβ induces IL-26 expression (31), while in fibroblasts, IL-1β and IL-17 induce IL-26 production. In colonic biopsies from IBD patients, Th17 cells (30), CD56 + NK cells (32), and CD68 + macrophages (32) have been reported to express IL-26. Thus, the cellular target of IL-26 in colonic biopsies is not precisely defined.

## IL-20 Cytokines have Pro- and Anti-Inflammatory Effects

The IL-20 cytokines IL-19, IL-20, IL-22, IL-24, and IL-26 are elevated in autoimmune/inflammatory diseases, such as in the skin of patients with psoriasis (18, 33, 34), in synovial fibroblasts, and macrophages of patients with rheumatoid arthritis (19, 35) and in patients with inflammatory bowel disease (20, 27, 30, 36–39). Likely, IL-20 cytokines have dual functions as they can exacerbate and attenuate inflammation depending on the tissue contexts by promoting wound healing, tissue protection, and regeneration (40, 41). Wound healing, tissue protection, and regeneration depend in part on activated STATs and expression of STAT1/STAT3 responsive genes, such as keratinocyte growth factor and epidermal growth factor (42, 43), expression of anti-apoptotic genes and mitogenic genes in hepatocytes (44), and protection of intestinal stem cells from apoptosis (45).

## IL-20 Cytokines in Esophageal Diseases

### Autoimmune/Inflammatory Diseases

Although the esophagus can be affected by inflammatory diseases, such as eosinophilic esophagitis (EoE), Crohn’s Disease, infectious diseases, or gastroesophageal reflux disease, the mucosal immune system of the esophagus is compared to the small and large intestine rather understudied. EoE, which has been increasingly diagnosed in the last decade, is thought to be a chronic food- and aeroallergen-mediated Th2 disease leading to tissue remodeling with strictures and stenosis (46). The Th2 cytokines IL-4 and IL-13 can induce IL-19 expression in keratinocytes and airway epithelial cells (47). Furthermore, increased expression of IL-19 has been observed in LPS-stimulated macrophages pretreated with IL-4 and IL-13 (48). Nevertheless, the contribution of IL-19, IL-20, and IL-24 in EoE have not been investigated yet.

### Infectious Diseases

IL-22 contributes to the clearance of oropharyngeal *Candida* infections (49) and is essential for the clearance in individuals and mouse models with an impaired Th1 response, whereas, loss of IL-22 may be compensated in individuals with an intact Th1 response (50). Because IL-26 is only expressed in humans and not in mice, the lack of animal models may in part explain why IL-26 has not been to our knowledge studied yet in contexts of esophageal diseases.

### Malignant Diseases of the Esophagus

Squamous cell carcinoma and adenocarcinoma can develop in the esophagus, but the contribution of IL-20 cytokine family members to the carcinogenesis is mainly unexplored so far. Increased expression of IL-19 and IL-20 in esophageal squamous cell carcinoma has been reported (22, 51), but potential mechanisms how members of the IL-20 cytokine family support the development of squamous cell carcinoma and adenocarcinoma in the esophagus is not known yet.

## IL-20 Cytokines in Gastric Diseases

### Autoimmune/Inflammatory Diseases

Autoimmune, chemical induced, and *Helicobacter pylori*-induced gastritis is the most common inflammatory conditions of the stomach. Contributions of the cytokines IL-19, IL-20, IL-24, and IL-26 for the development of autoimmune gastritis have not been reported yet.

### Infectious Gastritis

Significant increased IL-22 levels have been reported in *H. pylori* colonized individuals with peptic ulcer disease and gastritis (52). Interestingly, *H. pylori* can generate a niche, where *H. pylori* reside, by removing cholesterol from cell membranes and thereby preventing the formation of lipid rafts (53). These lipid rafts are required for the phosphorylation of JAK1, STAT1, and STAT3 in response to IFNγ and IL-22 signaling resulting in reduced production of antimicrobial peptides, such as β-defensin 3 (53). The immunization of *H. felis* with urease/cholera toxin increased IL-22 expression in the gastric mucosa (54). Inhibition of IL-22 with a neutralizing-antibody leads to impaired clearance of *H. felis* suggesting that IL-22 participates in control and clearance of *H. pylori* infections (54). Thus, IL-22 facilitates the clearance of *H. pylori*, but at the same time, *H. pylori* has developed sophisticated pathways by which *H. pylori* deprived itself of effects of IL-22.

### Malignant Gastric Diseases

IL-22 plays a role in host defense against *H. pylori*, a pathogen that supports the development of gastric carcinoma and mucosa-associated lymphoid tissue (MALT) lymphoma. Interestingly, single nucleotide polymorphisms in the *IL22* gene increases the risk for the development of MALT lymphoma threefold, but how these genotypes influence IL-22 expression and function is not known (55). Furthermore, IL-22 supports the migration and invasion of gastric cancer cells (56, 57). Gastric cancer cells express IL-22Ra and lymphatic invasion and tumor stage (57). Moreover, increased IL-22 producing T cell numbers in the tumor and the peripheral blood is associated with poor outcome (58, 59), indicating that IL-22 facilitates progression of gastric cancer.

The IL-20 cytokine IL-24 can induce apoptosis in gastric cancer cells and inhibits tumor angiogenesis *in vivo* in a chicken embryonic allantois model. Moreover, overexpression of IL-24 in the gastric cancer cell line SGC7901 sensitizes these cells to cisplatin, 5-fluorouracil, adriamycin, and methotrexate (60). By contrast, IL-26 promotes the proliferation of SGC7091 gastric cancer cells by modulating STAT1/STAT3 activation (61). A function of IL-19 and IL-20 in the development of gastric cancer is not reported in the literature so far.

Taken together, this indicates that the relevance of IL-20 cytokines for the development of gastric cancer is not well understood and that members of the IL-20 cytokine family may promote or protect from gastric cancer.

## IL-20 Cytokines in Intestinal Diseases

### Inflammatory Bowel Disease

**Figure 2:**
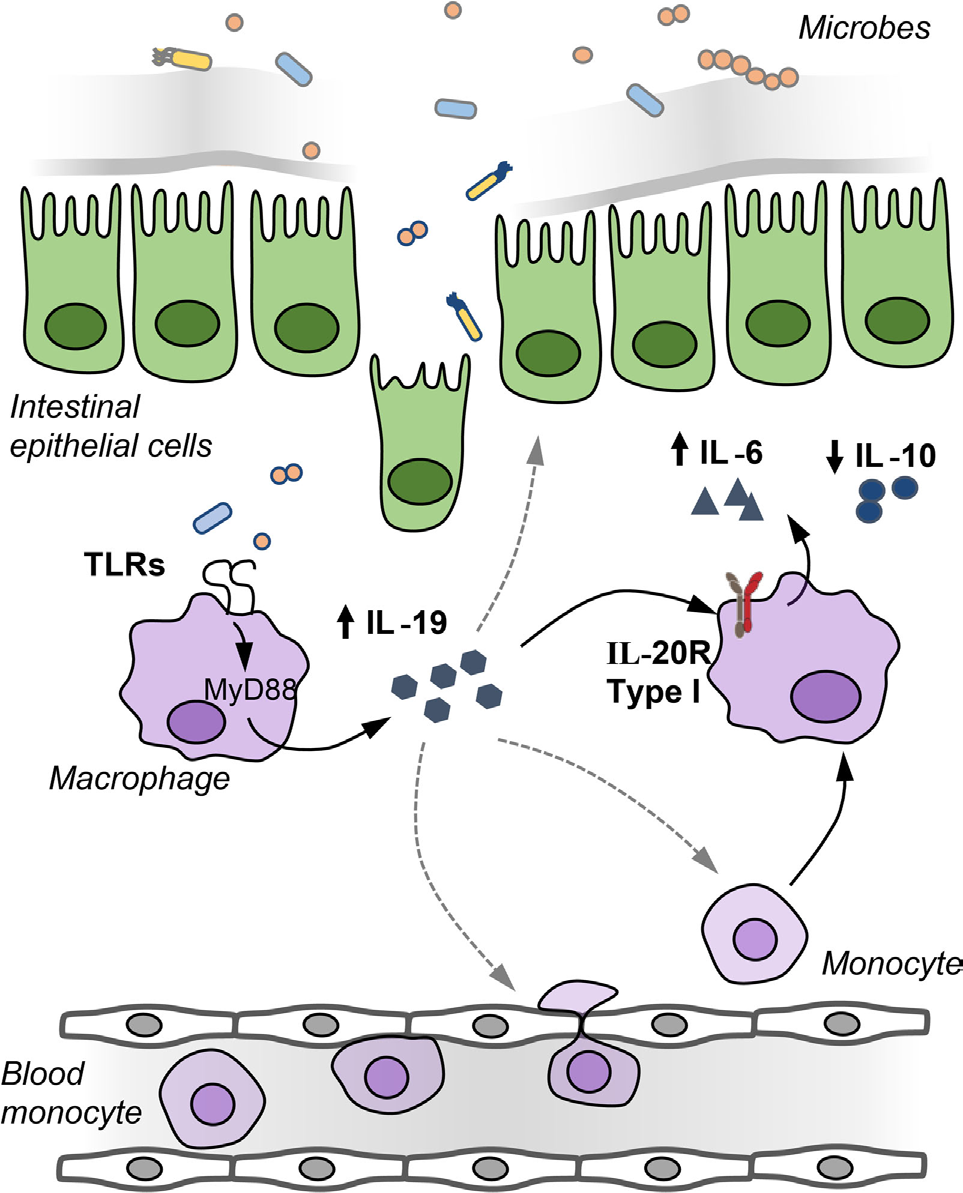
IL-19 regulates IL-6 production by macrophages in an autocrine manner. After barrier disruption, microbes or microbial-derived products invade the host to activate underlying monocytes and macrophages in the lamina propria to induce IL-19 production. IL-19 regulates in an autocrine manner the production of IL-6 by macrophages, IL-19 may recruit monocytes and macrophages to the inflamed gut, and IL-19 may affect the integrity of the intestinal epithelial barrier.

Although the expression of the type I and type II IL-20R is low in comparison to the IL-22 receptor (6, 9), there are some indications that the cytokines IL-19, IL-20, and IL-24 play a role in the pathogenesis of IBD. Increased expression of IL-19, IL-20, and IL-24 has been reported in biopsies of patients with ulcerative colitis and Crohn’s disease (20, 27, 39). In contrast, LPS-stimulated peripheral blood mononuclear cells (PBMCs) from Crohn’s patients produced less IL-19 compared to healthy controls (62). Furthermore, IL-19 suppressed the production of TNFα by PBMCs from healthy patients but not by PBMCs from Crohn’s disease patients (62). Moreover, single nucleotide polymorphisms in the *IL19* gene decrease the susceptibility to ulcerative colitis (63), while changes in the methylation status of the *IL19* gene locus have been associated with increased severity of Crohn’s disease (64). IL-19 is produced by macrophages, whereas subepithelial myofibroblasts have been identified as a source of IL-24, which induces the production of the mucins (mucin1, mucin3, and mucin4) (27). In colitis models, both accelerated and attenuated DSS colitis has been reported in two different IL-19 KO mouse strains (7, 17). Homozygous Il19-tdTomato reporter mice, in which tdTomato replaces exon 3 of the *Il19* gene tdTomato, showed attenuated DSS colitis associated with reduced IL-6 production in macrophages (Figure 2). Production of IL-19 by macrophages occurred after a breach of the intestinal barrier with DSS and required an intact bacterial flora because DSS treatment of gnotobiotic animals with a simplified oligo-mouse microbiota did not induce IL-19 production. IL-19 acts in an autocrine manner on mucosal macrophages, which acquire IL-20Rα during their differentiation into tissue macrophages to regulate the production of IL-6 as colonic macrophages express IL-20Rα in contrast to bone marrow-derived macrophages (7).

Increased expression of the cytokine IL-26 has been reported in biopsies of Crohn’s disease and ulcerative colitis patients (30, 32). IL-26 is produced by RORγt + Th17 cells in the mucosa and binds the IL-26 receptor expressed by subepithelial myofibroblasts and intestinal epithelial cells to induce the expression of the pro-inflammatory cytokines TNFα and IL-8 (30, 32). IL-26 is a cationic amphipathic protein that promotes the killing of extracellular bacteria by supporting the formation of membrane-pores and binding to bacterial DNA released from dying bacteria (31). This IL-26/DNA complex shuttles the DNA into myeloid cells, which activates the inflammasome in a STING-dependent manner (65). Interestingly, the translocation of bacterial DNA from the gut into the peripheral blood of Crohn’s disease patients leads to increased IL-26 concentrations. Single nucleotide polymorphism in the *IL26* gene results in reduced bacterial killing, elevated concentration of pro-inflammatory cytokines, and increased consumption of anti-TNF antibodies in Crohn’s disease patients with translocated bacterial DNA (66). Increased IL-26 concentration after translocation of bacterial-derived products from the gut into the host may serve as one line of an innate defense system to protect the host from invaded microbial products.

Elevated IL-22 expression was observed in biopsies of patients with Crohn’s disease and ulcerative colitis (36, 37). Overexpression of IL-22 in the colon of mice with a local gene delivery led to attenuated colitis associated with STAT3 activation and increased production of mucus-associated proteins by epithelial cells (5). Furthermore, IL-22 promotes wound healing *in vitro* and *in vivo* after the damage of the epithelial barrier (37, 67). T cells and type 3 innate lymphoid cells produce IL-22 during colitis in response to IL-23 and IL-1β (68). T cells in IBD patients also produce IL-22bp, which can neutralize soluble IL-22. Patients responding to treatment with anti-TNFα antibodies are characterized by reduced IL-22bp, while still producing the tissue-protective cytokine IL-22 (69). Perhaps, differential expression of IL-22bp and IL-22 by T cells in IBD patients receiving anti-TNFα antibodies may in part explain how anti-TNFα antibodies suppress active disease.

In summary, multiple functions of IL-20 cytokines in context of IBD have been described, such as the regulation of IL-6 production by macrophages through IL-19, recognition of bacterial DNA by IL-26, and wound healing by IL-22. However, whether a specific member of the IL-20 cytokine subfamily is a potential target for treatment of IBD requires further studies. These cytokines have pleiotropic functions that can protect from inflammation or facilitate colitis; thus, targeting this cytokine family for the treatment of IBD patients may be a challenge.

## Infectious Intestinal Diseases

Infection of mice with *Citrobacter rodentium* leads to increased IL-19, IL-20, and IL-24 expression. *C. rodentium* serves as a model for infections with attaching and effacing (A/E) bacterial pathogens, such as enteropathogenic *Escherichia coli* (6). Although *C. rodentium* infection increased IL-19, IL-20, and IL-24 expression, *C. rodentium* infected IL-20Rβ-deficient animals had comparable survival rates as wild-type animals (6).

However, the injection of a plasmid leading to the overexpression of IL-24 into animals prolonged survival rates after infection with *Salmonella typhimurium*. IL-24 attracted neutrophils and induced the production of IFNγ and IL-12 by neutrophils leading to the activation of CD8 T cells (70). Furthermore, the stimulation of human monocytes with *S. typhimurium* induced the expression of IL-20. When vasoactive intestinal peptide was added to the cultures a 16-fold decrease of IL-20 expression was noted (71), but the relevance of this observation has not been investigated *in vivo* yet.

Interestingly, IL-19 expression by M-CSF-differentiated bone marrow-derived macrophages was induced by stimulation with the TLR4 ligand LPS, the TLR9 ligand CpG, and the TLR2/6 ligand Pam2CSK4. Stimulation of macrophages with the TLR5 ligand flagellin or the TLR3 ligand poly (I:C) did not induce IL-19 expression, because M-CSF differentiated bone marrow-derived macrophages have a shallow TLR5 expression (7). Because TLR3 signals through TRIF and TLR4, TLR9, and the TLR2/6 require Myd88 for signaling (72), induction of IL-19 depends on Myd88 but not on TRIF. Microbes or their products are, therefore, strong inducers of the IL-20 cytokines.

Pivotal work has demonstrated that IL-22 is essential for the clearance of *C. rodentium* infection in mice. In part, macrophage-derived IL-1β and IL-23 facilitate IL-22 production by ILC3 during a *C. rodentium* infection (73–75). Production of IL-22 increases expression of antimicrobial peptides (e.g., RegIIIβ), production of mucins, and expression of fucosyltransferase 2 required for the fucosylation of epithelial cells (76). Fucosylated proteins are proteins to which the hexose deoxy fucose sugar units are added. Fucosylated proteins are shed into the intestinal lumen, where fucosylated proteins regulate the metabolism of microbes and microbial virulence genes (77). Furthermore, IL-22 regulates the expression of hemopexin, a hemoglobin scavenger, which partially regulates the systemic iron pool and thereby protects from infection (78). Controversially, pathogens are also able to utilize IL-22 to gain an advantage in the process of occupying a niche in the gastrointestinal tract. For example, *S. typhimurium* induced IL-22 expression leads to the production of the metal-chelating antimicrobial peptides calprotectin and lipocalin-2, which sequester iron. While *S. typhimurium* can adapt to iron starvation, iron starvation decreases the growth of commensal Enterobacteriaceae supporting an advantage for *S. typhimurium* (79).

## Colorectal Cancer

Because IL-24, which was initially named melanoma differentiation-associated gene-7, suppresses the growth and induces apoptosis of cancer cells (26), the expression of IL-24 in colorectal cancer patients has been determined. In a retrospective study with 96 rectal adenocarcinoma patients, 81 out of 90 patients expressed IL-24. Higher IL-24 expression was reported in well and moderate differentiated carcinomas, and expression of IL-24 negatively correlated with lymph node status as in patients with high IL-24 expression fewer lymph nodes were affected (80). Moreover, the expression of IL-24 was associated with the survival rate of the patients (81). Experimental studies with overexpression of IL-24 in cancer cell lines or studies, in which a small molecule stabilizes IL-24, suggested that IL-24 can potentially be used to enhance the efficacy of chemotherapies (81, 82).

IL-22 is involved in tissue repair and wound healing. Hernandez and colleagues recently excellently reviewed the importance of IL-22 for the development of colorectal cancer (83). Interestingly, both IL-22bp-deficient animals with elevated IL-22 and IL-22-deficient animals have increased tumor burden in colitis-associated cancer (CAC) model (84). Most likely, IL-22 has anti-inflammatory and antimicrobial effects that protect from cancer, while at the same time IL-22 supports the proliferation of epithelial cells promoting the development of cancer. The IL-20 cytokines IL-19, IL-20, IL-22, IL-24 and IL-26, IL-6, type I interferons, IL-5, and IL-10 signal all through STAT3 downstream of their receptors. Mice that lack STAT3 in epithelial cells develop fewer tumors in a CAC model and even fewer tumors compared to IL-6 deficient mice (85, 86). Thus, all individual cytokines that signal through STAT3 may contribute to the development of tumors. It would be interesting to compare tumor development in animals lacking specific IL-20 cytokines or individual receptor chains to STAT3-deficient animals to elucidate the contribution of single members of the IL-20 cytokine family to tumor formation.

## Conclusion

The increased interest in the IL-20 cytokine family revealed multiple sources and target cells indicating a wide variety of functions for IL-20 cytokines in the gastrointestinal tract in context of different diseases. The IL-20 cytokines IL-19, IL-20, IL-22, IL-24, and IL-26 are elevated in patients with IBD, gastrointestinal infections, and gastrointestinal cancers, of which IL-22 is the most intensively studied cytokine of the IL-20 cytokine family in contexts with gastrointestinal diseases by facilitating wound healing, tissue protection, and regeneration. Furthermore, IL-19-deficient animals develop attenuated DSS colitis that is induced by disruption of the epithelial barrier with DSS. Translocated bacterial-derived products induce IL-19 expression, which acts back on macrophages in an autocrine manner to regulate IL-6 production by macrophages. Future studies with a focus on single members and receptors of the IL-20 cytokine family are required to further understand their relevance for chronic inflammatory diseases, clearance of infectious diseases, and development of cancer in the gastrointestinal tract. Targeting individual members of the IL-20 cytokine family in mice may ultimately pave the way for this cytokine system as a target for therapy of gastrointestinal diseases.

## Author Contributions

JHN drafted and wrote the manuscript. PH reviewed the manuscript and gave suggestions. TK wrote the manuscript and designed the figures.

## Conflict of Interest Statement

The authors declare that the research was conducted in the absence of any commercial or financial relationships that could be construed as a potential conflict of interest.
